# Patients Undergoing Hip or Knee Arthroplasty in Poland Based on National Data—Challenge for Healthcare in Aging Society

**DOI:** 10.3390/healthcare9080924

**Published:** 2021-07-22

**Authors:** Mateusz Gajda, Agnieszka Pac, Barbara Gryglewska, Paulina Gajda, Anna Różańska, Jadwiga Wójkowska-Mach

**Affiliations:** 1 Doctoral School in Medical Sciences and Health Sciences, Jagiellonian University, Św. Łazarza 16 St., 31-530 Cracow, Poland; mgajda@doctoral.uj.edu.pl; 2 Department of Microbiology, Faculty of Medicine, Jagiellonian University Medical College, Czysta 18 St., 31-121 Cracow, Poland; mbmach@cyf-kr.edu.pl; 3 Department of Epidemiology and Preventive Medicine, Jagiellonian University Medical College, Mikołaja Kopernika 7A st., 33-332 Cracow, Poland; agnieszka.pac@uj.edu.pl (A.P.); paulina.gaca@wp.pl (P.G.); 4 Department of Internal Medicine and Gerontology, Jagiellonian University Medical College, Śniadeckich 10 St., 31-531 Cracow, Poland; barbara.gryglewska@uj.edu.pl

**Keywords:** knee arthroplasty, hip arthroplasty, aging society, orthopedics, healthcare, infection prevention

## Abstract

Arthroplasty procedures are more frequently performed due to their impact on the quality of life. The aim of this study was to evaluate and analyze the Polish national datasets and registries for hip and knee arthroplasty across Poland in order to describe and understand the challenges for healthcare in an aging society. The study included national data on 83,525 hip or knee arthroplasties performed in 2017. Of those, 78,388 (93.8%, 63.0% females) were primary operations: 66.6% underwent hip replacement surgery (HPRO, mean age 68.43 years, SD 11.9), and 5137 were secondary operations (females: 62.9%), with 75.2% of those being HPRO (mean age 69.0 years, SD 12.0). The mean age of the patients undergoing knee surgery (KPRO) was 68.50 years (SD 8.2). The majority (79.9%) were scheduled. The main reason for hospitalization was arthrosis (84.2% in total, HPRO—76.5%, and KPRO—99.5%), then trauma (15.1%; *p* < 0.001). In 5137 cases (6.2%, 62.9% females) in revision surgery group, 75.2% underwent HPRO (mean age 69.0 years; SD12.0), and 24.8% KPRO (mean age 68.0 years; SD 10.5). Similarly, 71.1% were scheduled. The main reason for hospitalization was complications (total—90.9%, HPRO—91.4%, and KPRO—89.4%) (*p* < 0.001). Comorbidities were present (over 80%) with the level of influenza, hepatitis B vaccination, and pre-hospital rehabilitation not exceeding 8% each in both groups. Due to the increasing age of patients, implicating comorbidities, there is a need for better preparation prior to surgery.

## 1. Introduction

Arthroplasty is a procedure performed mostly to improve joint functionality. The need for hip or knee arthroplasty usually arises from the impact of the damaged joint on the patients’ quality of life. In a study by Gajewski et al., on the basis of a questionnaire, the authors concluded that the most common localization of joint pain in patients over 53 years of age is the knee joint (43.7% of respondents on average), followed by a similar frequency of shoulder and hip joints (33.3% of respondents on average) [1].

Currently, total hip replacement (HPRO) or knee replacement (KPRO) is the gold standard in the treatment of osteoarthritis, rheumatological degeneration, or trauma. These procedures have become the most frequently performed procedures in the field of the musculoskeletal system [2], which results, directly from the impact on patients’ lives, in both improvement of mobility and quality of life—and the problem of the length of functionality of endoprostheses depends on the age of patients, which significantly shortens with time [3]. In addition, other authors note the growing need for arthroplasty in the other limb or other joints on the same side due to gait disturbances and an increased risk of degeneration of one’s own joint after primary surgery [4].

Poland is an aging country, with the current share of people aged 60+ reaching 25% of the population [5]. In the period from 2000 to 2018, life expectancy for both men and women increased even further. However, almost 70% of older people, more women than men, suffer from long-term health problems or chronic diseases lasting 6 months or more. Moreover, almost half of the elderly show limitations in the activities of daily living. Disability largely depends on the increasing incidence of degenerative joint changes developing with age. That is why the advancements in orthopedics and the growing possibilities of arthroplasty of the knee and hip joint provide an opportunity to improve the quality of life, mobility and maintaining independence, especially for the elderly in Poland. Therefore, knee and hip arthroplasties are among the surgical procedures recommended by the European Centre for Disease Prevention and Control (ECDC) for targeted surveillance in the HAI-Net project [6]. However, such an approach to infection control concerning this type of surgery is not very popular in Poland. Targeted, continuous surveillance of hip and knee arthroplasty was implemented by a few hospitals participating in the Polish Society of Hospital Infections program using ECDC patient-based protocol of surgical site infections (SSI) registration. The results obtained in this project revealed substantial and higher SSI rates in Poland than in other European countries [7]. Ziółkowski et al., in a study based on laboratory microbiological data surveillance, found even six times higher rates of SSI than the average in ECDC reports [8]. There can be numerous and varying factors influencing the epidemiology of SSI in hip and knee arthroplasty. No detailed analysis of the population of patients undergoing such procedures has been conducted in Poland so far, because no targeted prospective studies were performed in this area. National Health Fund payer data base of refunded procedures is the only, but a valuable, source of data in this case. The aim of this study was to evaluate and analyze the Polish national datasets and registries for hip and knee arthroplasty across Poland in order to describe and understand the challenges for healthcare in an aging society.

## 2. Materials and Methods

The analysis was carried out on the basis of an anonymized database, including 83,525 patients over 18 years of age undergoing hip or knee arthroplasty. The data include information reported to the National Health Fund (NFZ) by various reporting systems and relate to operations that took place between 1 January 2017 and 31 December 2017, and because the analyses involved checking each patient’s fate in the perioperative period (before and after discharge or before surgery), some of the data also come from 2016 and 2018. The data include only the procedures performed within the general health care system (no data from private centers). The current analysis employs data concerning adults who underwent hip or knee prosthesis, identified on the basis of ICD-9 codes: 81.51–81.55, 00.7 or 00.8.

The data were analyzed separately for patients undergoing primary and revision surgery, divided into hip and knee surgery in both analyzed groups. Then the data were compiled by analyzing the groups in relation to the primary and revision surgery.

The obtained collection in the scope of this publication included: demographic information (age, place of residence), selected elements of preparation for surgical procedures (vaccinations, rehabilitation, medications taken), and information on endoprosthesis (divided into hip and knee joints). A detailed description of the data provided can be found in the Appendix A. Factors included in the analysis were determined by data gathered by the National Health Fund (NFZ).

Due to the anonymization of the obtained data, at the stage of cleaning the database and determining the relevant data from the researchers’ point of view, several assumptions were made to facilitate grouping and statistical analysis of the existing data:

- The patients’ place of residence, assigned to the urban or rural category, was determined with precision as to whether the place of residence belongs to the municipal or rural commune, respectively;
- The burden of patients with particular disease entities at the preoperative stage was assessed on the basis of information on the use of specific drug groups in the period of one year before the date of the procedure, hence multimorbidity was defined as the use of drugs from at least two different groups according to the anatomical therapeutic chemical (ATC) classification code, with a note that only prescription drugs were considered;
- Taking drugs from 5 or more ATC-code groups was a criterion for polytherapy—with a note that only prescription drugs were considered;
- The indication for influenza vaccination was a procedure planned in the infectious season; with regard to vaccination for hepatitis B, we included vaccination in any time prior to surgery, provided that the vaccines were purchased on prescription;
- Preoperative rehabilitation concerned 90 days preceding the surgery, provided that it was a reimbursed service.

This analysis was performed separately for primary surgery and revision surgery. The analysis took into account the available data for the state before surgery (current, index hospitalization) or for the day of surgery (age). The group of patients undergoing hip replacement arthroplasty was compared to those undergoing knee replacement surgery.

Data on the frequency of arthroplasty in the Polish population are presented in the form of prevalence rates per 100,000 people in the appropriate sex and age groups (5-year age ranges) in the population according to the data, as of 30 June 2017 [9].

### 2.1. Statistical Analysis

In the statistical analysis of the collected material, relative and absolute frequencies were used for nominal variables and the mean value with standard deviation for quantitative variables (age). Chi2 test and Student’s *t*-test were used to compare the groups of patients (hip vs. knee arthroplasty). The analysis was carried out in the SPSS Statistical Package for the Social Sciences (STATISTICS 24, Armonk, NY, USA). In all analyses, the significance level was α = 0.05.

### 2.2. Ethics

This study was approved by the Bioethics Committee of the Jagiellonian University No. 1072.6120.149.2020 of 25 June 2020.

## 3. Results

In the database provided, 93.8% (n = 78,388) of cases were primary operations, among which HPRO was dominant (66.6% vs. 33.4%). Of all patients, 49,394 (63.0%) were women and 28,994 (37.0%) were men. The mean age of patients was 68.4 years (SD 11.9) for HPRO (70.9, SD 11.8) for women, 65.0 (SD 11.5) years for men and 68.5 years (SD 8.2) for KPRO (69.2, SD 7.9) for women, 66.6 (SD 8.8) years for men. Both types of procedures were performed more often in women (HPRO 57.9%; KPRO 73.1%) (Table 1).

**Figure 1:**
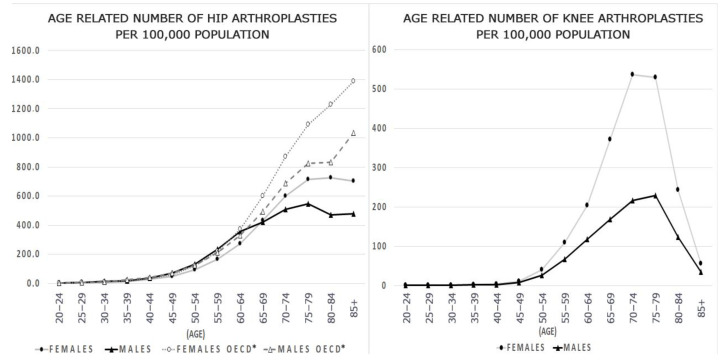
Number of hip and knee replacement procedures per 100,000 people in Poland and OECD. * OECD data-2008 or latest year available.

Both among women and men in the population undergoing hip arthroplasty, we observe an increase in prevalence from the age of 45 years, with a peak around the age of 75 years. At the age of about 65 years, the frequency of procedures among women begins to outweigh those performed in men, reaching a maximum of 726.6 per 100,000. For men, the prevalence was lower by nearly 200 cases per 100,000. For the knee joint, we have similarly observed an increase in the prevalence from about 45 years old with the overwhelming frequency of procedures among women reaching a peak of 537.1 per 100,000 at the age of 70–80 years old. For men, the peak is observed in similar years, but with a much lower frequency, reaching 229.0 per 100,000. The age-specific rates of hip and knee replacement per 100,000 females and per 100,000 males follow similar trends, as shown in Figure 1.

The vast majority of patients (79.9%) were admitted electively, and the main reason for hospitalization was joint degeneration (total—84.2%, HPRO—76.5%, and KPRO—99.5%), followed by trauma in 15.1% of patients (*p* < 0.001). Even though the predominant number of admissions were scheduled, only 0.5% of patients before HPRO and 0.6% before KPRO were vaccinated against influenza, despite indications for 54.4% and 56.0%, respectively. A similarly low percentage of people were vaccinated against hepatitis B—around 5% for both types of surgery.

In terms of preoperative rehabilitation, 4090 (5.2%) patients were covered by this type of care. In the conducted analysis, the place of residence did not show any statistically significant differences, despite a greater number of operations among people living in urban communes.

Chronic polytherapy was found in 48.7% of patients with the predominance of HPRO (52.2% vs. 42%). The vast majority of patients (total 81.2%, KPRO 87.3%, HPRO 78.1%) qualified for the multimorbidity criterion. The majority of the admitted patients were those taking drugs from the groups intended for cardiovascular diseases (71.1%) and gastrointestinal diseases, including diabetes (53.4%).

Revision procedures were performed in 5137 patients, most often HPRO (75.2% vs. 24.8%), 3230 (total 62.9%, HPRO 60.6%; KPRO 69.9%) in women, and 1907 (37.1%) in men. The mean patient age was 69.0 years (SD 12.0) for HPRO (70.8 (SD 11.5) for women, 66.1 (SD 12.1) years for men, and 68.0 years (SD 10.5) for KPRO (69.3, SD 10.0) for women, 65.1 (SD 11.2) years for men (Table 2). The vast majority of patients (71.1%) were admitted as scheduled and the main reason for hospitalization were complications (ICD-10: T84) of the previous procedure (total—90.9%, HPRO—91.4%, and KPRO—89.4%) (*p* < 0.001).

As in the group of patients undergoing primary surgery, only 0.4% of patients before HPRO and 0.6% before KPRO were vaccinated against influenza, with indications for such vaccination for 53.5% and 52.7%, respectively. Vaccination against hepatitis B was performed for about 3% of both types of treatments. In terms of preoperative rehabilitation, only 389 (7.6%) patients were covered by this type of care, which was related to postoperative rehabilitation after primary surgery. Similarly, no significant differences regarding the place of residence were found, despite a greater number of operations among people living in urban communes.

In the group of patients undergoing revision surgery, chronic polytherapy was found in 61.0% of patients, more often in patients requiring KPRO (68.8% vs. 58.5%). Additionally, the majority of patients (total 87.2%, KPRO 91.8%, HPRO 85.7%) qualified for the multi-morbidity criterion, reached, together with polytherapy, a higher percentage in the revision group compared to the original group. The majority of patients were taking drugs from the groups for cardiovascular diseases (70.9%) and gastrointestinal diseases, including diabetes (58.9%), but also diseases of the muscular (65.3%) and nervous (57.2%) systems.

Comparing the groups of primary and revision surgeries in terms of statistical significance, we are observing the frequency of preoperative rehabilitation in favor of revision surgery (7.6% vs. 5.2%) and the level of vaccination against hepatitis B in favor of primary surgery (5.1% vs. 3.0%). In the remaining variables, the groups achieved statistical homogeneity (Table 3).

When analyzing the burden of patients on admission to the hospital, the greater burden of patients undergoing revision surgery in terms of all statistically significant results is noticeable. This directly implies greater multi-disease among this group (87.2% vs. 81.2%). Hip joint reoperation was significantly more frequent (75.2% vs. 66.6%). Detailed data are presented in Table 4.

## 4. Discussion

Hip or knee arthroplasty procedures constitute a significant percentage of orthopedic surgeries [2] due to the improvement of both the quality of life and the motor performance of the operated persons. OECD data indicate that in recent years the access to this type of treatment has significantly improved, increasing their number by about 7% in 2000–2009, and, according to recent data, the increase in Poland in 2017 amounted to nearly 20% [10]. Therefore, hip arthroplasty procedures, in addition to the cesarean section and transurethral prostatectomy, have been recognized as the most common and most significant operations in improving patients’ quality of life [11].

Additionally, in the analyses of the prevention and control of healthcare-associated infections (HAIs), such treatments are among the most important for continuous surveillance, both at the national level, e.g., in Great Britain [12], and internationally, e.g., in the Healthcare-Associated Infections Surveillance Network (HAI-Net) program which is a European network for surveillance of HAIs, coordinated by the European Centre for Disease Prevention and Control (ECDC). Participation in HAI-Net is voluntary and confidential for European hospitals [13].

According to Singh, the situation in the United States and other European countries shows an increase in the number of both primary and revision surgeries for the hip and knee (from 10% to 60% over several or over a dozen years) [2]. Meanwhile, in Poland, despite the general increase in the number of procedures performed, knee revision surgeries remain unchanged at about 1200 cases, while the number of hip revision surgeries decreased slightly (4507 in 2016, compared to 3861 for the current data). Nevertheless, based on the data obtained, it is difficult to explain this opposite tendency for revision procedures. Perhaps it is dictated by the dependence solely on data from facilities contracted by the National Health Fund, which is one of the limitations of this study.

In Poland, 83,525 arthroplasties were performed in the analyzed period, which, according to the data prepared by OECD, would place Poland in 3rd place in terms of the number of procedures performed, behind Germany and Great Britain. According to the data obtained, the procedures are indeed most often performed in geriatric patients over 65 years of age, and this happens despite the fact that total HPRO in older patients was associated with being more likely to post-operative complications, admission to the ICU, discharge to a skilled care facility, and longer hospital stay, older patients seemed to have a similar improvement in quality of life [14]. This is in line with the general trend, but on the other hand, Polish patients are about 3–4 years younger than patients undergoing the same treatments in highly developed countries, i.e., according to ECDC data for 2017, the average age for HPRO was 72 years, while for KPRO 70 years). The cause of this condition may be the greater number of urgent patients in Poland than in the ECDC reports. Probably the main reason for this is the high proportion of admissions of patients with trauma (10.1% vs. 20.1%), requiring urgent arthroplasty and in younger patients, while in the EU countries, the osteoporotic degeneration of the joint is probably a much more frequent cause of the surgery [13].

The above observation regarding the growing trend concerning the age of patients is confirmed by OECD reports, indicating the highest share among operated people aged 75–80 years old; interestingly, in Poland, we do not observe a second peak in the frequency of these procedures after the age of 85 [11]. On the other hand, the total number of hip arthroplasty procedures is much lower than the OECD average from 2008 data (approximately 1400/100,000 vs. 726/100,000 for women and 1000/100,000 vs. 540/100,000 for men). Age-standardized rates vary from 50 per 100,000 in Portugal (for both men and women) to 161 per 100,000 in Norway (for women) and 167 in Switzerland (for men) [11]. In terms of knee arthroplasty, this frequency does not differ from the curves observed in other countries with an increase after 50 years of age, a peak around 75–80 years of age, and then a decrease [15]. Gender differences, which are similar in our and other studies for both primary and revision surgeries, are mainly due to the general tendency for osteoporosis to be overwhelmingly prevalent in women of all ages [16,17]. The dominance of women was also found in the studied Polish population, especially for knee arthroplasty—similarly to the study from South Korea [18]. Kim et al. explained this fact with more severe degeneration of the joint, higher BMI, and loads among women, as evidenced by slightly higher percentages of people taking medications from given groups among women (43.1% vs. 37.6% on average). Smaller differences, but also with a predominance of women in both types of treatments, were observed by Abdelaal et al. assessing global changes in the field of arthroplasty procedures [19].

The average age of patients operated on will probably increase due to the aging of the population. Nevertheless, due to the improvement of the quality of life and general efficiency, endoprosthesis procedures should be performed regardless of age, and only on the basis of a comprehensive geriatric assessment and the assessment of fragility syndrome among the elderly, which is especially confirmed in oncological operations [20]. The oldest people in our data to undergo hip arthroplasty were 104-year-olds, and 95-year-olds for the knee. Moreover, the age in the revision group was similar to the primary group, and we also expect an increase in this group which is associated with a higher risk of complications. It is related to technical aspects, but also to increasing comorbidities which are even higher for the revision group despite similar ages as in our study (87.2% vs. 81.2%) [21].

In our study, the vast majority of patients were admitted for joint degeneration, which is consistent with the expected age-related cause of osteoporosis [19]. Worldwide data show that osteoporosis affects 22.1% of women and 6.1% of men over 50 years of age. The number of people suffering from osteoporosis in Poland in 2018 was 2.1 million, of which 1.7 million were women [22]. In 2018, a total of 331,800 patients were granted consultations in the field of osteoporosis, of which almost 80% were provided in an osteoporosis (43.9%) or rheumatological (35.0%) clinic. In the field of diagnostic tests, 176,000 tests were performed in the same year. In Poland, over 80,000 knee and hip joint prostheses are implanted each year, which, compared to the number of sick and diagnosed patients, may indicate insufficient screening among these patients. In our database, most of the people undergoing procedures came from municipalities, which may mean easier access to specialist clinics (especially strictly osteoporotic ones) or densitometric clinics. On the other hand, in Poland, cities are inhabited by 60.1% of the population, while rural areas are inhabited by 39.9% [23], this corresponds to the general structure of the operated patients, therefore it is in conflict with the conclusion that the inhabitants of rural areas had worse access to specialist care. In the analyzed cases, the reasons for primary and revision operations are significantly different (degeneration in 84.2% for primary and complications in 90.9% for revision surgery). Nevertheless, complications resulting from osteoporosis may be also responsible for some of the revision surgeries. This conclusion is supported by the studies conducted by Namba et al. in which bisphosphonates reduced the risk of revision surgeries [24].

On the basis of the obtained data, the insufficient preparation of patients for arthroplasty procedures is extremely puzzling, despite the fact that 80% of the operations are scheduled. The level of influenza vaccination of patients at about 0.5% is definitely unsatisfactory, taking into account the level of respiratory morbidity estimated on the basis of the groups of drugs used to the extent of 15–20% in the studied population. Tartof et al. point out that, although there are only a few studies assessing the effectiveness of influenza vaccination in the prophylaxis of pneumonia in the perioperative period, they confirm the safety of such a procedure, contrary to some opinions [25]. Currently, there are no studies showing an increased risk for patients from periprocedural vaccination. The low percentage of patients reported as vaccinated against HBV is also striking, despite the fact that in many units this vaccination is the basic element of preparation for the procedure. However, the data do not include people who may have been vaccinated during previously scheduled hospitalizations, and some procedures were performed urgently, preventing vaccination. Nevertheless, some patients were vaccinated only before revision surgeries, which may indicate a previous lack of vaccination, despite the previously existing indications for its implementation. However, the nature of the obtained database is at this point another limitation of the study, which does not allow for a clear answer to the question about the cause of such a state.

In the reported data, the burden on the revision group was greater in terms of gastrointestinal diseases, including diabetes, as well as hematopoietic, musculoskeletal, nervous, and respiratory systems. This is important because some of the complications, which are the main cause of reoperation, can be avoided by appropriate preventive measures—such as motor and respiratory rehabilitation. Coudeyre et al. point out that the use of preoperative rehabilitation shortens the hospital stay and reduces postoperative costs [26]. Such a procedure also ensures a greater chance of returning directly to the place of residence, bypassing rehabilitation departments, etc. Unfortunately, in the population studied, a very low share of pre-rehabilitation in pre-operative preparation was found: only 5–7% of people were prepared for the procedure despite the fact that it could significantly contribute to effective upright standing and mobilization and rehabilitation of patients. Vodička et al. seems to provide further arguments pointing to deficits in flexors and extensors as predictors of arthroplasty. In their study, an imbalance reduced by 8% decreases the probability of THA by the same percentage value [27]. Therefore, data obtained in other studies indicate a beneficial effect of preoperative rehabilitation on the reduction in the risk of the need to perform the procedure itself with an adequately early response or reduction in the hospitalization period, its costs, and faster return to daily activity.

In order to reduce the risk of postoperative complications, the employment of not only pre-operative rehabilitation, in the sense of improving the overall fitness and development of muscle strength [27], is advisable but also, e.g., psychological health and social functioning should be worked on [28,29].

In the analyzed population, the number of people meeting the assumed criteria of polypharmacy or multi-disease is alarming. However, this is not an unexpected result due to the advancement of people over 65 in terms of both types of arthroplasty, but on the other hand, the analyzed data may be underestimated due to the assessment of the above-mentioned parameters based on the number of groups of drugs taken. Mazya et al. point to the problem of fragility syndrome among geriatric patients and assess it as a critical point in the approach to this group [30]. Despite this, the correct overall geriatric assessment proposed in the authors’ study indicates the possibility of adequate early intervention and improvement of the general condition of patients [30]. Due to the characteristics of the population, the appropriate preparation of patients for procedures is the greatest challenge in the group of patients undergoing arthroplasty.

Undoubtedly, the limitation of this study is the access only to information resulting from reports for the payer to the National Health Fund (NFZ), while the course of treatment and the overall effect of the surgery is influenced by a number of factors that are not accessible to the people involved in this study. Some of the data were determined secondarily according to the assumptions developed in the research methodology due to the lack of direct data describing a given feature of the respondents.

## 5. Conclusions

To conclude, in the Polish population, despite the existing indications, the vast majority of patients are insufficiently prepared for surgery. Despite modern medical knowledge regarding postoperative complications—including pulmonary and cardiac complications—the level of influenza vaccination is very low. This is important because the average age of the people undergoing surgery will increase in line with the forecasts of other researchers, and one of the indications for influenza vaccination in Poland is the older age. Similarly, a very low level of periprocedural rehabilitation seems unacceptable due to the potential goal of treatment, which is to improve the quality of life and fitness of patients. In light of the obtained results supported by other studies, it would be advisable to implement appropriate rehabilitation from the very first indications of joint dysfunction. This intervention will reduce the number of procedures performed, and will prepare the people who need to undergo them for postoperative return to normal motor function. It is extremely important due to the percentage of patients with deficits in the area of the musculoskeletal and nervous systems, as shown in this article.

Due to the low percentage of people vaccinated against influenza, it would be recommended to require vaccination during the period of morbidity, reducing the number of pulmonary complications after procedures, which are a high risk, especially among the elderly.

Due to the numerous limitations resulting from retrospective falls on the basis of the reported data, it would be necessary to conduct a prospective study assessing patient management and prophylaxis, risk factors, and the end result of the procedure. Nevertheless, the authors of the article, on the basis of the currently available data, will try to assess, in particular, complications after arthroplasty procedures in order to set out actions aimed at preventing their occurrence in the postoperative period.

## Figures and Tables

**Table 1:** Characteristics of the population for primary surgery.

Characteristics of the Study Group	Hip, HPRO n = 52,207 (66.6%)	Knee, KPRO n = 26,181 (33.4%)	Total, n = 78,388
**Age by categories, years, n; %**			
<65 years	18,574 (35.6%)	7620 (29.1%)	26,194 (33.4%)
≥65 years	33,633 (64.4%)	18,561 (70.9%)	52,194 (66.6%)
**Gender, n; %**			
Female	30,249 (57.9%)	19,145 (73.1%)	49,394 (63.0%)
Male	21,958 (42.1%)	7036 (26.9%)	28,994 (37.0%)
**Place of residence, n; %**			
City	30,260 (58.2%)	15,272 (58.5%)	45,532 (58.3%)
City-countryside/countryside	21,763 (41.8%)	10,832 (41.5%)	32,595 (41.7%)
**Rehabilitation prior to surgery, n; %**			
Yes	2554 (4.9%)	1536 (5.9%)	4090 (5.2%)
**Influenza vaccination, n; %**			
Yes	262 (0.5%)	160 (0.6%)	422 (0.5%)
No indications	23,789 (45.6%)	11,507 (44.0%)	35,296 (45.0%)
**HBV vaccination,** **n; %**			
Yes	2619 (5.0%)	1348 (5.1%)	3967 (5.1%)

Missing data in the variable place of residence n = 261. Abbreviations: HBV, hepatitis B virus; HPRO, hip replacement; KPRO, knee replacement.

**Table 2:** Characteristics of the population for revision surgery.

Characteristics of the Study Group	Hip, HPRO n = 3861 (75.2%)	Knee, KPRO n = 1276 (24.8%)	Total, n = 5137
**Age by categories, years, n; %**			
<65 years	1248 (32.3%)	413 (32.4%)	1661 (32.3%)
≥65 years	2613 (67.7%)	863 (67.6%)	3476 (67.7%)
**Gender, n; %**			
Female	2338 (60.6%)	892 (69.9%)	3230 (62.9%)
Male	1523 (39.4%)	384 (30.1%)	1907 (37.1%)
**Place of residence, n; %**			
City	2281 (59.4%)	770 (60.6%)	3051 (59.7%)
City-countryside/countryside	1561 (40.6%)	501 (39.4%)	2062 (40.3%)
**Rehabilitation prior to surgery, n; %**			
Yes	265 (6.9%)	124 (9.7%)	389 (7.6%)
**Influenza vaccination** **, n; %**			
Yes	16 (0.4%)	8 (0.6%)	24 (0.5%)
No indications	1796 (46.5%)	604 (47.3%)	2400 (46.7%)
**HBV vaccination, n; %**			
Yes	109 (2.8%)	43 (3.4%)	152 (3.0%)

Missing data in the variable place of residence n = 24. Abbreviations: HBV, hepatitis B virus; HPRO, hip replacement; KPRO, knee replacement.

**Table 3:** Comparison of primary and revision groups.

Characteristics of the Study Group	Primary n = 78,388 (93.8%)	Revision n = 5137 (6.2%)	Total, n = 83,525	*p*-Value
**Age by categories, years, n; %**				
<65 years	26,194 (33.4%)	1661 (32.3%)	27,855 (33.3%)	0.115
≥65 years	52,194 (66.6%)	3476 (67.7%)	55,670 (66.7%)	
**Gender, n; %**				
Female	49,394 (63.0%)	3230 (62.9%)	52,624 (63.0%)	0.858
Male	28,994 (37.0%)	1907 (37.1%)	30,901 (37.0%)	
**Place of residence, n; %**				
City	45,532 (58.3%)	3051 (59.7%)	48,583 (58.4%)	0.052
City-countryside/countryside	32,595 (41.7%)	2062 (40.3%)	34,657 (41.6%)	
**Rehabilitation prior to surgery, n; %**				
Yes	4090 (5.2%)	389 (7.6%)	4479 (5.4%)	<0.001
**Influenza vaccination, n; %**				
Yes	422 (0.5%)	24 (0.5%)	446 (0.5%)	0.054
No indications	35,296 (45.0%)	2400 (46.7%)	37,696 (45.1%)	
**HBV vaccination, n; %**				
Yes	3967 (5.1%)	152 (3.0%)	4119 (4.9%)	<0.001

Missing data in the variable place of residence n = 285. Abbreviations: HBV, hepatitis B virus.

**Table 4:** The burden of patients divided into the primary and revision group.

Characteristics of the Study Group	Primary n = 78,388 (93.8%)	Revision n = 5137 (6.2%)	Total, n = 83,525	*p*-Value
**Disease of the digestive system (including diabetes), n; %**				
Yes	41,879 (53.4%)	3025 (58.9%)	44,904 (53.8%)	<0.001
**Disease of the hematopoietic system, n; %**				
Yes	19,286 (24.6%)	2605 (50.7%)	21,891 (26.2%)	<0.001
**Disease of the cardiovascular system, n; %**				
Yes	55,723 (71.1%)	3643 (70.9%)	59,366 (71.1%)	0.808
**Disease of the hormonal system, n; %**				
Yes	18,790 (24.0%)	1245 (24.2%)	20,035 (24.0%)	0.678
**Neoplasms, n; %**				
Yes	3683 (4.7%)	310 (6.0%)	3993 (4.8%)	<0.001
**Disease of the musculo-skeletal system, n; %**				
Yes	47,926 (61.1%)	3355 (65.3%)	51,281 (61.4%)	<0.001
**Disease of the nervous system, n; %**				
Yes	35,882 (45.8%)	2936 (57.2%)	38,818 (46.5%)	<0.001
**Disease of the respiratory system, n; %**				
Yes	12,363 (15.8%)	910 (17.7%)	13,273 (15.9%)	<0.001
**Type of admission, n; %**				
Urgent	15,676 (20.0%)	1473 (28.9%)	17,149 (20.6%)	<0.001
Planned	62,598 (80.0%)	3624 (71.1%)	66,222 (79.4%)	
**Reason for surgery, n; %**				
Other	544 (0.7%)	51 (1.0%)	595 (0.7%)	<0.001
Complications	66 (0.1%)	4670 (90.9%)	4736 (5.7%)	
Trauma	11,803 (15.1%)	164 (3.2%)	11,967 (14.3%)	
Degeneration	65,975 (84.2%)	252 (4.9%)	66,227 (79.3%)	
**Multimorbidity, n; %**				
Yes	63626 (81.2%)	4481 (87.2%)	68,107 (81.5%)	<0.001
**Operated joint, n; %**				
Hip	52,207 (66.6%)	3861 (75.2%)	56,068 (67.1%)	<0.001
Knee	26,181 (33.4%)	1276 (24.8%)	27,457 (32.9%)	

Missing data in the variable type of admission n = 154.

## Data Availability

The datasets generated and analyzed during the current study are available from the corresponding author on reasonable request.

## Abbreviations

ATC	Anatomical-therapeutic-chemical classification,
HBV	Hepatitis B Virus
NFZ	National Health Found
TISS	Therapeutic intervention scoring system

## Appendix A

Data provided in the form of a collection from the National Health Fund.

**1. Patient data:**
(a) Gender
(b) Age
(c) Place of residence: village, town below 100,000 residents, a city with over 100,000 residents
(d) Was the patient vaccinated (up to definition) in the year before the operation due to:
i. HBV; vaccination at any time (depending on the archival data of the National Health Fund), at least 14 days before the procedure
ii. flu:
(1) surgery performed during the flu season, i.e. on October 1–March 31: vaccination should take place on September 1 of the preceding year—up to 7 days before surgery
(2) if the operation took place in the off-season: i.e. April 1–September 30—there were no indications for vaccination
(e) Rehabilitation within 90 days prior to surgery, as defined in ICD-9
(f) Whether the patient suffered from selected chronic diseases, defined on the basis of purchased drugs, acc. ACT code, respectively in groups:
i. drugs used in diseases of the digestive system, including diabetes: A02BC, A10 A10B, A10B
ii. drugs acting on the hematopoietic system: B01: B01AA/B01AB/B01AC/B01AD/B01AE/B01AF/B01AX, B03
iii. drugs in cardiovascular diseases: C01/C02/C03/C04/C05/C06/C07/C08/C09/C010,
iv. hormonal drugs: G03, H01/H02/H03/H04/H05
v. anti-cancer drugs: L01/L02/L03/L04
vi. drugs acting on the musculoskeletal system: M01/M02/M03/M04/M05/M09
vii. drugs acting on the nervous system: N01/N02/N03/N04 N04/N06/N07
viii. drugs affecting the respiratory system: R01/R02/R03/R04/R05/R06/R07
ix. polytherapy: taking 5 drugs or more regardless of the number of groups from how many of the above groups does the patient take drugs: i.e. 0 or 2, 3 etc.
**2. Entity carrying out the procedure:**
(a) Is it a learning unit
(b) Number of arthroplasty: knees/hips that the unit performed in the year before surgery
(c) Distance from the patient’s place of residence
**3. Information about arthroplasty:**
(a) primary/revision/revision without replacing elements
(b) partial/total
(c) cemented/cementless
**4. Data on hospitalization related to arthroplasty:**
(a) Mode of admission (emergency or scheduled)
(b) Reason for hospitalization according to ICD-10 codes
(c) Time from admission to hospital to surgery
(d) Was the procedures defined in accordance with ICD-9 during the operation
(e) Time from surgery to discharge from the department where the operation was performed
(f) What was the ward to which the patient was transferred from the ward where the operation was performed (according to Part VIII of the departmental code)
i. Length of stay in this ward
ii. If it was an anaesthesiology and intensive care unit, the maximum TISS value from this stay
(g) Time from discharge from the ward to discharge from the hospital
(h) Was hospitalization fatal?
**5. Have drugs from the following groups been prescribed on discharge (date of order equal to the date of discharge)**
(a) defined as redemption, according to ATC code:
i. appropriate drug, according to ATC code: M01/M02/M03/M04/M05/M06/M07/M08/M09, H05
ii. all antibiotics, i.e. all J01 drugs
**6. Infections after surgery**
(a) Was the patient treated within 90 days of surgery for infection and inflammation caused by an internal joint prosthesis (ICD-10: T84.5)
(b) Was the patient provided services for lower respiratory tract, urinary tract, digestive and other infections defined by the ICD-10 within 30 days of the operation?
**7. For all patients, data on post-operative hospitalization services**
(a) Have any inpatient rehabilitation services been provided within 42 days of the operation?
(b) Have any outpatient rehabilitation services been provided within 42 days of the operation?
(c) Did the patient receive benefits at care and treatment facility (National Health Fund(NFZ): based on concluded contracts) within 90 days from the operation
(d) Has the patient used a long-term care nurse (NFZ: on the basis of concluded contracts) within 90 days from the operation
**8. For all patients, if the patient died during hospitalization within 120 days of surgery**
**9. For patients who received services for the reasons listed in point 6, data on services after hospitalization related to the operation (up to 120 days from the date of surgery)**
(a) Admission to the ward
i. Reason for admission according to ICD-10
ii. Distance from the patient’s place of residence
iii. Department to which the patient was admitted: conservative/interventional/anesthesiology and intensive care unit (if it was an anesthesiology and intensive care unit, the maximum TISS value from this stay)
iv. Was the prosthesis revision performed (definition according to ICD-9 codes)
v. Is it the same center that performed the arthroplasty?
vi. length of hospitalization
vii. whether secondary hospitalization resulted in the patient’s death
viii. Hospitalization cost
(b) Admissions to the Emergency Department
i. Reason
ii. Is it the same site that performed the operation?
(c) Outpatient treatment or primary health care
i. Reason f
ii. Is it the same site that performed the operation?
iii. Was it an internal medicine practitioner/geriatrician/orthopaedist/general practitioner, defined on the basis of part VIII of the departmental code
(d) Prescriptions after surgery (order date greater than the date of discharge, up to 30 days from the date of surgery): each prescription with a direct reference to the procedure performed, i.e. demographic details, details of the prescription
i. whether there was a drug from a given group, only antibiotics: all drugs from the J01 group with the specification of the substance (according to codes e.g. J01CR02)
ii. the amount of the substance (or information on the number of packages with the dose in the package)
iii. date of issue of the prescription
iv. demographic stratification of patients taking into account groups of patients classified according to the operating procedure.
